# Pregnancy and Susceptibility to Infectious Diseases

**DOI:** 10.1155/2013/752852

**Published:** 2013-07-07

**Authors:** Elisabeth Sappenfield, Denise J. Jamieson, Athena P. Kourtis

**Affiliations:** Division of Reproductive Health, National Center for Chronic Disease Prevention and Health Promotion, Centers for Disease Control and Prevention, Atlanta, GA 30341, USA

## Abstract

To summarize the literature regarding susceptibility of pregnant women to infectious diseases and severity of resulting disease, we conducted a review using a PubMed search and other strategies. Studies were included if they reported information on infection risk or disease outcome in pregnant women. In all, 1454 abstracts were reviewed, and a total of 85 studies were included. Data were extracted regarding number of cases in pregnant women, rates of infection, risk factors for disease severity or complications, and maternal outcomes. The evidence indicates that pregnancy is associated with increased severity of some infectious diseases, such as influenza, malaria, hepatitis E, and herpes simplex virus (HSV) infection (risk for dissemination/hepatitis); there is also some evidence for increased severity of measles and smallpox. Disease severity seems higher with advanced pregnancy. Pregnant women may be more susceptible to acquisition of malaria, HIV infection, and listeriosis, although the evidence is limited. These results reinforce the importance of infection prevention as well as of early identification and treatment of suspected influenza, malaria, hepatitis E, and HSV disease during pregnancy.

## 1. Introduction

Pregnancy is often thought to be associated with increased susceptibility to infection. For example, during the 19th and early 20th century, pregnancy was thought to have a deleterious effect on the course of tuberculosis, so much so that therapeutic abortion was recommended in pregnant women with tuberculosis [1]. However, during the second half of the 20th century, after radiography became available, it became clear that the extent of disease, radiographic pattern, and individual susceptibility were more important than pregnancy itself in determining the course and prognosis of the disease. After the advent of effective chemotherapy, pregnant women with tuberculosis have the same generally good prognosis as nonpregnant women.

In the 1950s, the transplant immunologist Peter Medawar proposed that during pregnancy there is a general maternal immune suppression in order to assure tolerance of the semiallogeneic fetus [2–4]. Our understanding of the immune alterations that occur during pregnancy has evolved considerably since Medawar's time to include more complex theories of immune alteration. There is evidence that adaptive immune responses are weakened, potentially explaining reduced viral clearance [4–7]. Evidence also suggests a boosted innate response [4, 6, 8], which may represent a compensatory immune mechanism to protect the pregnant mother and the fetus and which may imply decreased susceptibility to initial infection.

## 2. Objective 

We reviewed the available evidence related to infection risk and clinical course/maternal outcomes during pregnancy. The hypotheses tested were whether pregnancy is associated with increased susceptibility to infection or with increased severity of infectious diseases.

## 3. Methods for Review

 We searched PubMED (L.S.) in July of 2012 and reviewed the literature that pertained to pregnancy, infection, disease susceptibility, or severity of illness. The terms used were “pregnancy,” “pregnant,” “gestation,” “infection,” “severity,” “susceptibility,” and “pregnancy outcome.” Since we only intended to focus on maternal, and not infant, outcomes, and we focused on humans, we excluded the terms “fetus,” “infant,” “neonate,” “children,” or “veterinary.” Additionally, we examined the reference lists of all identified articles for further relevant resources and hand-searched the personal files of the authors of this review. We aimed to include studies that reported information on infection risk or disease outcome in pregnant women.

## 4. Results

**Figure 1:**
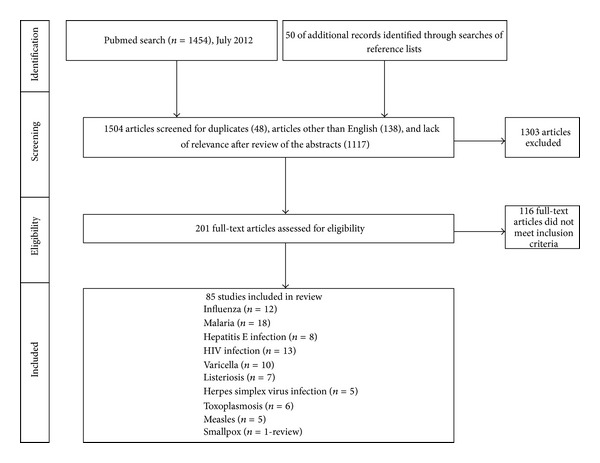
Study selection.

The PubMed searches that included the term “susceptibility” identified 136 journal articles, and those including “severity” identified 1318 journal articles. Fifty additional articles were found through manual searches of reference lists. Articles were then excluded for lack of relevance after review of the titles and abstracts (1117), duplicates (48), and language other than English (138). Infectious diseases for which two or fewer articles were identified (31) were not included. Only studies that reported information on infection risk or disease outcome in pregnant women were included. This resulted in 85 articles included (original articles and reviews) (Figure 1). Information was extracted from these publications by two of the authors (Elisabeth Sappenfield and Athena Kourtis), synthesized, and presented per infectious agent.

## 5. Influenza

There is considerable evidence that pregnant women are at increased risk for more severe illness from influenza infection. Cardiopulmonary adaptive changes occurring in pregnancy, such as increased cardiac rate and stroke volume and reduced pulmonary residual capacity, may increase the risk of hypoxemia and contribute to the observed increased severity of influenza. During the pandemic of 1918, the maternal mortality rate was 27% (50% when complicated by pneumonia), and during the pandemic of 1957, 50% of fatalities among reproductive-age women occurred among those pregnant ([9, 10] reviewed in [11]). Surveillance data from the 2009 influenza A pandemic showed that 509 of 788 influenza cases of pregnant women reported to the CDC from April through August 2009 were hospitalized. Among the 509 hospitalized pregnant women, 115 (22.6%) were admitted to the intensive care unit and 30 (6%) died [10]. Severity of illness was higher for women in their third trimester, with a higher proportion of intensive care unit (ICU) admissions among those presenting in the third trimester, although severe illness was reported among women presenting in all three trimesters [10]. Primary viral pneumonia was also more frequent in pregnant women [10]. In the United States, 5% of all pandemic influenza deaths were among pregnant women although pregnant women represent only about 1% of the US population [10]. A higher rate of hospital admissions and medical encounters for pregnant women with confirmed or suspected influenza versus the general population and greater severity of disease during late pregnancy was also found in interpandemic periods ([12–14] reviewed in [15]). In contrast to influenza A, pregnancy did not seem to be associated with complicated influenza B infection [16]. With regards to case fatality rates, a retrospective study of pregnant and postpartum women from a low income country found an association of H1N1 infection in pregnancy with increase in mortality rate (25% versus 8% in nonpregnant women) [17]; studies from Canada and Lithuania, however, did not observe increased case fatality in pregnant women [18, 19], likely the effect of early antiviral therapy.

## 6. Malaria

*Plasmodium Falciparum (P. falciparum)* is the most studied of all species of malaria parasites that infect humans. In pregnancy, the harmful effects of *P. falciparum* have been long recognized (maternal anemia, low birth weight, intrauterine growth restriction, and preterm birth, among others) [20]. *P. falciparum* is the only species known to sequester in the placenta, and this placental sequestration is believed to be the cause of many of the manifestations of falciparum malaria during pregnancy. Most evidence comes from Africa, where *P. falciparum* infections predominate. The frequency of *P. falciparum* malaria is greater in pregnant than nonpregnant women in highly endemic areas [21–24]; this represents, in essence, an expression of disease severity, since in such settings women have already been exposed to malaria parasites. McGregor found the prevalence of malarial parasitemia in females aged 15–45 years to be significantly higher in pregnant than in nonpregnant women in their surveys from 1961 to 1975 in rural Gambia [25]. A slightly higher prevalence of parasitemia in pregnant than nonpregnant women of childbearing age (36.6% versus 28.8%, PR 1.27 [1.05–1.53]) was found in the Congo using DNA-based methods [26]. Women in their third trimester were at highest risk for clinical malaria in some [27] but not all studies [28]. Many studies have reported decreasing malaria susceptibility with increasing parity [25, 28]. This is most pronounced in areas of high malaria endemicity [29]. Young maternal age may be an additional and independent risk factor of malaria in pregnancy.

*P. vivax*, the second most prevalent malarial infection, has been associated with low birth weight and maternal anemia in studies from India, Thailand, and Indonesia [30–32]. It also seems to cause more severe disease in pregnancy. Like with *P. falciparum* malaria, primigravidae were also more susceptible to *P. vivax* than multigravidae [30].

Susceptibility to and severity of malaria are determined by the level of immunity, which depends mainly on the intensity and stability of malaria transmission. In areas of low or unstable transmission, infected women become symptomatic and if untreated, the infection can progress rapidly to complications with a high case fatality rate. In reports from India, at least one quarter of maternal deaths were attributable to malaria [33]. Pregnant women have a 3-fold risk of severe malaria than nonpregnant women; a median mortality rate of 39% was reported in studies from the Asia-Pacific region. Maternal mortality has also been reported with *P. vivax* infection [33]. In areas of high transmission, most women harboring parasites do not present with symptoms. It had been thought that in such areas severe malaria and related mortality in pregnancy were rare [20, 29, 34]. Recent evidence suggests, however, that the impact of malaria on maternal deaths in sub-Saharan Africa has been underestimated and that malaria in pregnancy may be an important direct cause of maternal complications and mortality [20, 24, 29, 35]. Of those symptomatic the majority are primigravidae; multigravidae women from highly endemic areas rarely present with clinical signs or symptoms of malaria, even in the presence of high parasitemia [24, 29]. Malaria in subsequent pregnancies is typically less severe than in the first pregnancy [36]. The reasons for this observation are unclear, although the predominant theory suggests that *P. falciparum* parasites accumulate selectively in the placenta, by interacting with syncytiotrophoblastic chondroitin sulphate A (CSA), if the parasites express particular antigenic variants [37]. Women who experience a malaria episode from CSA-binding parasites during their first pregnancy lack immunity to the antigenic variants presented by these strains (even though they may be immune to other variants—that bind endothelial receptors—from previous infections) and are highly susceptible to the new infection. 

 Regardless of endemicity or level of immunity, maternal malaria causes anemia in the mother, which can, if severe, contribute to maternal mortality. 

## 7. Listeriosis

Primarily a food-borne pathogen, *Listeria monocytogenes* can contaminate a variety of raw foods such as uncooked meats, vegetables, unpasteurized milk, and soft cheeses. Listeria infections most commonly occur during the third trimester and are rare earlier in pregnancy [38]. Infection may be asymptomatic or may present as a flu-like illness; severe infection is rare, with no maternal deaths reported among pregnant hospitalized women [39]. Listeriosis has however a predilection for the placentofetal unit and, depending on the stage of pregnancy, can lead to pregnancy loss, stillbirth, premature birth, or serious neonatal disease. Pregnancy accounted for one-third of all cases of culture-confirmed listeriosis in a laboratory surveillance system over a 2-year period [40]. An active surveillance study of culture-confirmed listeriosis in the US in 1986 found that 67 of 246 cases were in pregnant women with 22% of perinatal cases resulting in stillbirths and neonatal deaths [38]. Active population-based surveillance determined that 17% of 762 listeriosis cases reported in 10 US geographic sites between 2004–2009 were in pregnant women [41]. Hispanic women were particularly at risk [41–43], perhaps due to their dietary practices. It has been estimated that invasive listeriosis associated with pregnancy is from 13 to over 100 times as common as in the general population [41, 42, 44]. However, over 50% of pregnancy cases were associated with a neonatal case [41], suggesting that fetal and neonatal sequelae of listeriosis often bring to light pregnancy cases that might otherwise go unrecognized and may thus bias estimates of pregnancy-conferred risk.

## 8. Hepatitis E Virus Infection

Hepatitis E virus (HEV) infection exhibits increased severity of illness in pregnant women, with a high mortality rate in the third trimester [45, 46]. In areas of high endemicity, such as in India, South East Asia, Middle East, and Africa, HEV infection can be a major cause of maternal and fetal death. The pathophysiology of this increased mortality is not well understood. 

In a review of all consecutive cases of acute liver failure from 1989 to 1996 in an endemic area in India, 49 of 83 women of childbearing age were pregnant (33 in their third trimester); in 47 of these pregnant women liver failure was due to HEV infection [47]. A study from India reported that one-third of 28 pregnant women with HEV infection had a severe form of hepatitis, and the case fatality rate of HEV among pregnant women was 27% [48]. In another study, 12 of 28 (43%) women with symptomatic HEV infection in pregnancy developed fulminant hepatic failure; 2 died before delivery and 1 died postpartum [48]. A review indicated the case-fatality rate of HEV among pregnant women to be between 15% and 25%, compared with 0.5% to 4% in the population overall [49]. In a study of 220 consecutive pregnant women presenting with jaundice caused by acute viral hepatitis, fulminant hepatic failure was more common (relative risk of 2.7), and maternal mortality was greater (relative risk of 6.0) in HEV-infected women [50]. Another study in India examined 50 pregnant and 50 nonpregnant women with fulminant hepatic failure: a higher seropositivity rate for HEV (30% versus 14%) and a significantly higher mortality rate among those HEV-positive (66% versus 24%) were found in pregnant compared with nonpregnant women [51]. Another Indian study confirmed an increased incidence of fulminant hepatic failure among HEV-infected pregnant women; however, the incidence of HEV in pregnant women was not significantly different from that in nonpregnant women [52].

## 9. Herpes Simplex Virus (HSV) Infection

The clinical manifestations of recurrent genital herpes (frequency of subclinical versus clinical infection, duration of lesions, pain, and constitutional symptoms) are similar in pregnant and nonpregnant women [53]. Recurrences increase in frequency over the course of pregnancy [53]. HSV disseminated disease and hepatitis are rare in adults and are mostly seen in immunocompromised patients. Primary HSV infection in pregnant women confers a higher risk for dissemination and hepatitis, particularly during the third trimester. HSV hepatitis may result from HSV 1 or 2 and can also be a result of not only primary but latent infection as well. To date, 27 cases of HSV hepatitis during pregnancy have been reported [54]. A review of the literature [55] reported that pregnancy represented the second largest group of adults with disseminated HSV; mean gestational age at presentation was 30.6 weeks, and case fatality was 39% for both mother and fetus/neonate [55]. Chase et al. found 8 pregnant women among 35 well-documented cases of HSV hepatitis in adults (23% of all cases) [56]. Another review found that 21 of 56 cases of HSV hepatitis in adults were pregnant women; however, the mortality rate (43%) was lower compared with that of nonpregnant patients (approaching 100%), attributed by the authors to the severe immunocompromising conditions in most of the nonpregnant patients, as well as to treatment with acyclovir in more of the pregnant patients in this series [57].

## 10. Toxoplasmosis

*Toxoplasma gondii* is a coccidian protozoon transmitted by consuming undercooked meat or by ingesting water or soil contaminated by cat feces. The annual seroconversion rates of pregnant women for *Toxoplasma gondii* in different geographic regions range from 0.6% to 1.5% [58–60]. Antoniou et al. found the seroconversion rate to be between 0.1% and 3.3% among 5,532 pregnant women in Greece over a 5-year period [61]. Avelino et al. studied prospectively the incidence of seroconversion among 3564 women of childbearing age between 1997 and 1999 in Brazil. 1114 of the women were seronegative upon screening, thus susceptible to infection; almost half of them were pregnant. The women were followed with subsequent serologic tests for various lengths of time ranging from 6 months to 1 year. The authors reported that 66% of women who seroconverted were pregnant; pregnant women had a 2.2 times higher risk of seroconverting. However, no information on whether frequency of testing for pregnant and nonpregnant women was standardized is given, and this study reported the highest ever rate of seroconversion (8.6%) [62]. Another study found no association between pregnancy and susceptibility to toxoplasmosis [63]. A study in England found a much lower rate for seroconversion in 1621 stored antenatal paired serum specimens (0.03%) than was expected for women in England (0.18%–0.95%), suggesting a decreasing incidence and arguing against a higher risk in pregnant than nonpregnant women [64]. Thus, there is little evidence for increased susceptibility of pregnant women to acquisition of toxoplasmosis. Age, location, and individual risk factors (exposures) seem to be the more important determinants of susceptibility to infection than pregnancy itself.

## 11. Human Immunodeficiency Virus (HIV) Infection

There is some evidence that HIV incidence may be particularly high among pregnant women in many settings [65–67]. A 2-fold risk of HIV infection during pregnancy was shown in a prospective study of 1085 serodiscordant couples with HIV-susceptible female partners in Kenya; this risk seemed however to be explained in part by behavior and other factors (higher frequency of unprotected sexual activity among women who become pregnant) [68]. Similar estimates were reported in another study from Uganda [69]. Other studies from African settings have not confirmed a higher risk of HIV acquisition in pregnancy [70, 71]. Biologic factors such as hormonal changes during pregnancy, including increased concentrations of progesterone, have been hypothesized to alter local immune responses and the genital tract mucosa to increase the risk of HIV infection during pregnancy [69].

Pregnancy does not seem to result in acceleration of HIV disease in most HIV infected women. Large studies from the US and Europe did not show acceleration of HIV replication or disease progression in pregnancy [72–75]. Some data from developing countries suggested progression of HIV disease in pregnancy [76]. Differences in the impact of pregnancy on HIV disease across geographic areas may reflect differences in potential confounding factors, such as poverty, nutrition, other concurrent infections, and greater likelihood of advanced HIV disease at the time of pregnancy. Cellular immunity and CD4+ lymphocyte levels are expected to decline during pregnancy in all women but eventually return to prepregnancy levels [77].

## 12. Varicella

Primary varicella has a 25-fold risk for complications in otherwise healthy adults, compared with children [78], with varicella pneumonia being the most frequent serious complication in adults. It is uncommon during pregnancy, estimated from 0.8 to 7/10,000 [79–81], which is not different from estimates in all adults [82, 83]. Early reports postulated that pregnancy, particularly in the third trimester, is a risk factor for severe chickenpox. This evidence was mostly based on reviews of published case reports or small case series [78, 84, 85]. In a 1990 review of 34 published cases of varicella pneumonia, the mortality of pregnant women was 35% [78], higher than that of nonpregnant adults (11.4% in another study) [86]. In contrast, Paryani and Arvin reported a varicella pneumonia rate of 9% with one death and one requiring ventilator support among 43 pregnant women with varicella [85]. Esmonde et al. reviewed reports on varicella pneumonia in adults published from 1965 to 1989; of the 99 cases, 46 were women, and of these 28 were pregnant (21 in the last trimester), suggesting an increased rate of varicella pneumonia in pregnancy; however, mortality (10%) was not greater in nonpregnant adults [84].

More recent studies do not support higher varicella severity during pregnancy. In a New York City survey, only one of 144 women died [79]. In a study from Great Britain and Germany that followed 1373 women with chickenpox during pregnancy, no maternal deaths were reported [87]. More recent studies have the benefit of earlier diagnosis and treatment, better supportive care, and use of antiviral agents that have improved the outlook of varicella pneumonia in general. 

## 13. Measles

There is some evidence, mostly from small case series, that measles displays increased severity during pregnancy. A measles outbreak in Greenland in 1951, before a measles vaccine was available, showed higher mortality in pregnant, compared with nonpregnant women [88]. From 1988 to 1991, when there was a resurgence of measles in the US, a number of pregnant women developed measles. Of 13 such women hospitalized in Houston, 54% had respiratory complications requiring admission to an intensive care unit and one died [89]. The cause of severe disease was thought to be primary measles pneumonia. Chiba et al. described the results of a measles outbreak in Japan in 2000-2001; eight women were infected during pregnancy. Three of the 4 cases who acquired the infection before 24 weeks ended in spontaneous abortions or stillbirth related to severe maternal disease [90]. In a report of 38 women with measles during pregnancy, 60% were hospitalized and 6% had pneumonia; 3% died [91]. In a study from Saudi Arabia, 80% of 40 women with measles during pregnancy were hospitalized, 4 with severe pneumonia, but none died; no such complications were seen among 37 nonpregnant women with measles [92]. 

## 14. Smallpox

A review of the literature on the outcomes of smallpox during pregnancy, spanning the 19th and 20th centuries, yielded 1,074 pregnant patients, with 368 (34%) maternal deaths; case fatality was highest during the third trimester and higher in unvaccinated women [93]. In some, but not all, of the studies reviewed in this report, the case fatality in pregnancy was higher than that in nonpregnant adults. The proportion of miscarriage or premature birth was 39.9%; risk for these outcomes did not vary by trimester of pregnancy [93]. Given that smallpox has been successfully eradicated since the 1970s, the available evidence may be subject to limitations of earlier investigations, including incomplete reporting and variable extent of supportive care.

## 15. Comment

Immune alterations with advancing pregnancy may impair pathogen clearance, resulting in increased severity of disease for several pathogens. The evidence is most convincing for influenza, malaria, hepatitis E, and HSV infection; more limited evidence is available for measles and smallpox. The evidence does not support increased severity of varicella during pregnancy. The evidence for increased susceptibility to initial infection during pregnancy is not as clear-cut. Pregnant women may be more susceptible to acquisition of listeriosis and HIV, although the maternal outcomes are not more severe; this conclusion is based on limited information. Toxoplasmosis does not seem more frequent in pregnancy. 

Vaccination during pregnancy is a strategy that has proven safe and effective for a number of infectious agents. Its beneficial effects may not be limited to the mother, but, by reducing fetal and placental inflammation, may have potential long-term benefits for the infant. Development of vaccines against other relevant pathogens, such as HSV or malaria, will be important. Early identification and appropriate treatment of infectious diseases during pregnancy are also important in preventing complications and maternal mortality. Additional research regarding safety of new vaccines and therapies during pregnancy will be crucial in increasing their uptake in this population.
